# Bilateral Nasolabial Cysts: A Rare Clinical Entity

**DOI:** 10.7759/cureus.106866

**Published:** 2026-04-11

**Authors:** Deeksha Shetty, Akhil P Singh, Saumyata Neeraj, Dharmendra Kumar

**Affiliations:** 1 Otolaryngology - Head and Neck Surgery, Sarojini Naidu Medical College, Agra, IND

**Keywords:** bilateral, nasoalveolar cyst, nasolabial cysts, non-odontogenic, oral surgery

## Abstract

Nasolabial cysts are rare non-odontogenic, extraosseous cystic lesions that commonly present as unilateral submucosal masses in the nasolabial fold, accounting for 0.7% of all non-odontogenic cysts. These lesions are a form of retention cyst. Bilateral presentations are extremely rare. They are typically slow-growing, painless swellings that cause cosmetic deformity and nasal obstruction. Diagnosis is largely clinical, supplemented by radiological and histopathological evaluation. They are managed surgically. We report a case of a young female who presented with a history of gradual, painless upper lip swelling and bilateral nasal obstruction following trauma. Clinical examination and radiological findings were indicative of bilateral nasolabial cysts. The patient underwent excision of the bilateral cysts, and histopathological examination confirmed the diagnosis.

## Introduction

Nasolabial cysts were initially reported by Zuckerkandl in 1882 [1]. These are infrequent developmental lesions located in the area of the ala nasi along the nasolabial folds, with no odontogenic origin, comprising less than 1% of all maxillary cysts [2,3]. These are classified as retention cysts and are now believed to arise following trauma or infection, from previously dormant epithelial tissue that develops into a cystic structure [4]. Clinically, these cysts present as unilateral painless swellings; bilateral presentation occurs in approximately 10% of cases and shows a female preponderance [5]. They cause facial disfigurement, especially when present bilaterally. Diagnosis is largely clinical, supplemented by radiological and histopathological examination. We discuss a case of a rare occurrence of bilateral nasolabial cysts on either side of the midline in a young female following an episode of trauma in the form of a fall on the face.

## Case presentation

**Figure 1:**
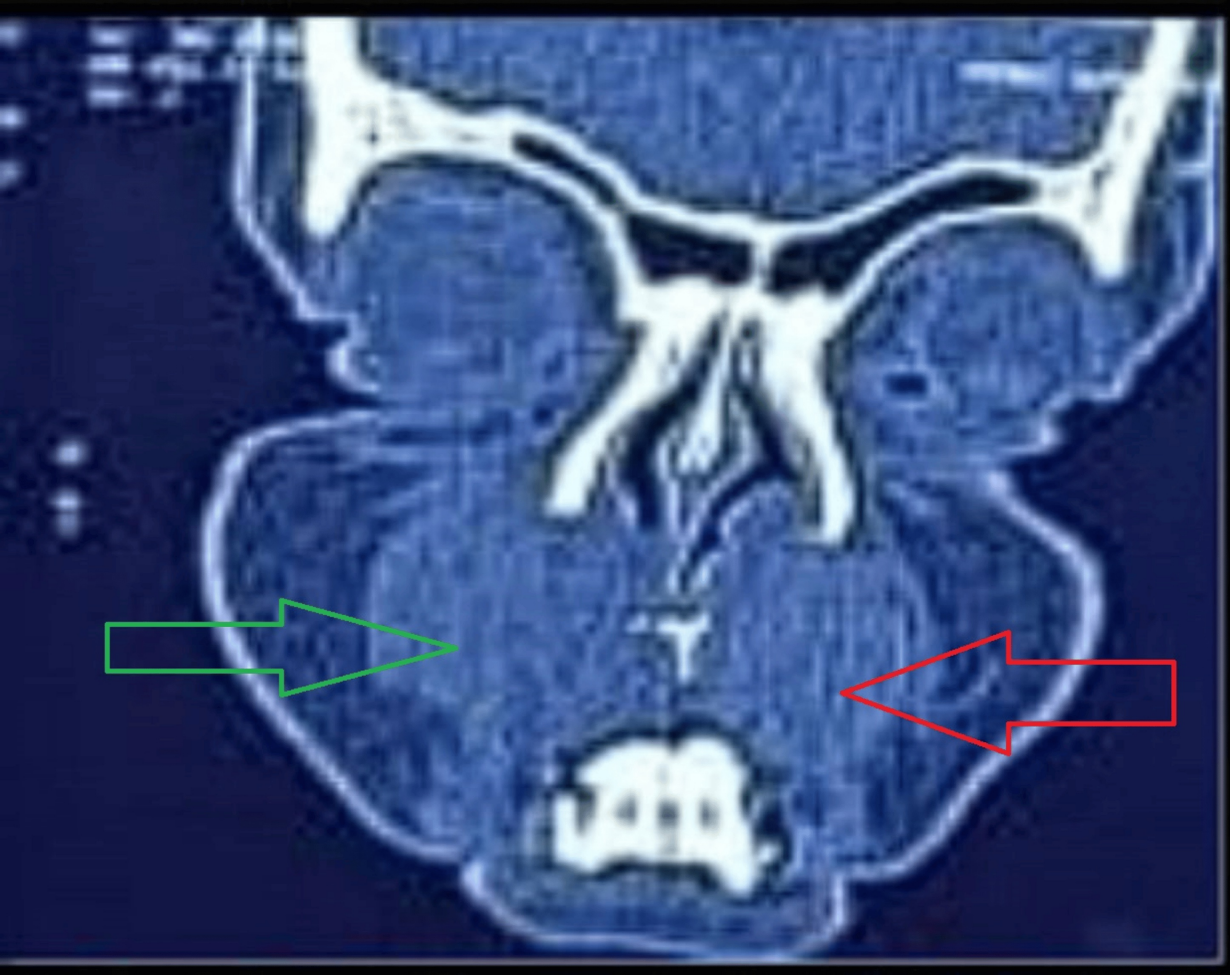
Non-contrast CT findings Two well-demarcated homogenous lesions in the bilateral nasolabial region. The green arrow shows a lesion measuring 2.6 x 2.7 x 3.4 cm on the right side, and the red arrow shows a lesion measuring 2.2 x 2.3 x 2.9 cm on the left side CT: computed tomography

A 30-year-old female presented with a 15-day history of gradual, painless upper lip swelling and bilateral nasal obstruction following trauma in the form of a fall over the face. Examination revealed a smooth, cystic, non-tender mass in the maxillary labial vestibule with obliteration of both nasolabial folds and bulging into the nasal floor. She denied epistaxis, nasal discharge, diplopia, or facial pain. Dental examination revealed no abnormality. Non-contrast CT showed two well-demarcated homogenous lesions in the bilateral nasolabial region measuring 2.6 x 2.7 x 3.4 cm on the right side and 2.2 x 2.3 x 2.9 cm on the left side, causing smooth scalloping of the underlying gingival surface of the alveolar process of the maxilla (Figure 1).

**Figure 2:**
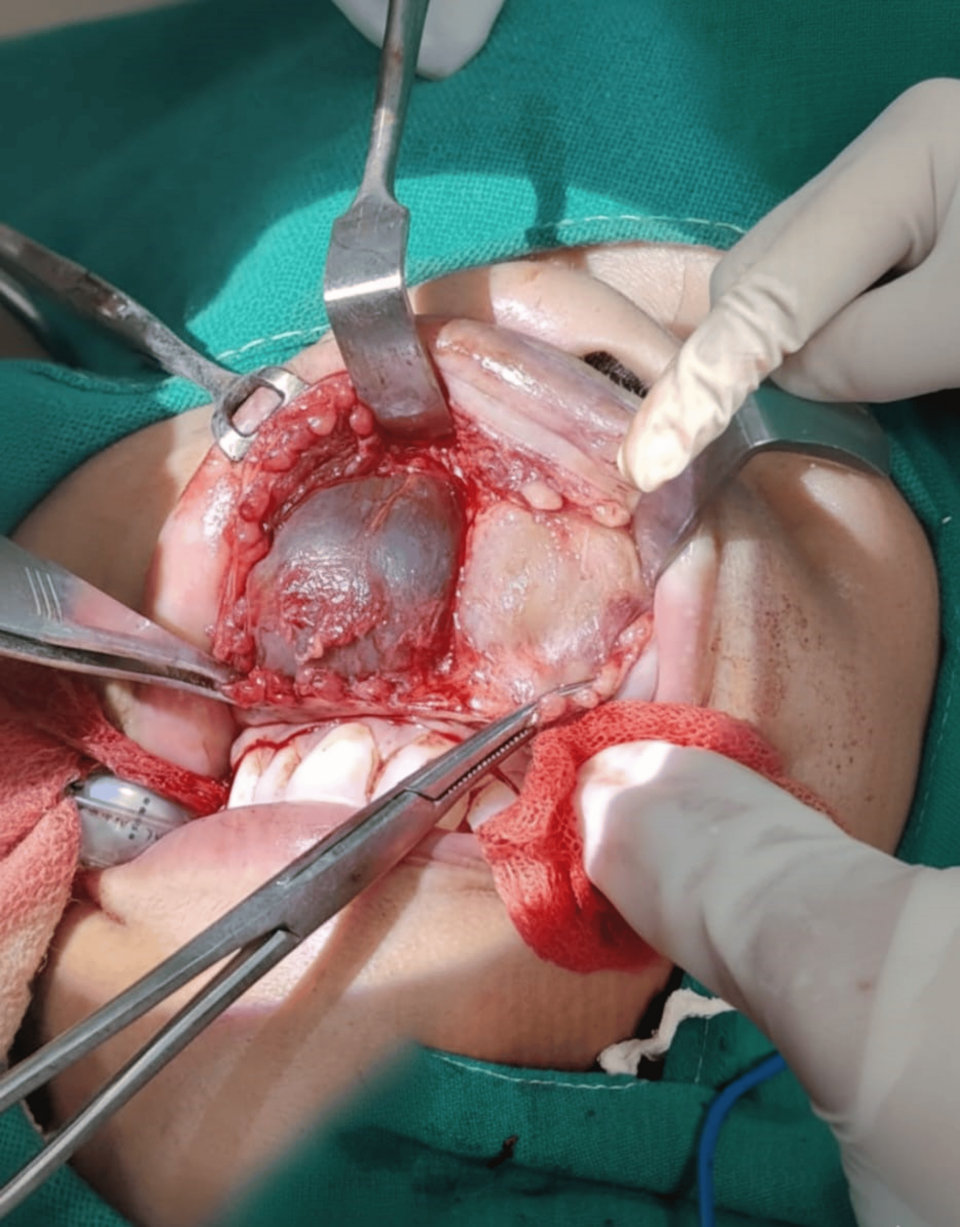
Intraoperative image showing two cysts separated by a bony septum

Cytology demonstrated straw-colored fluid with few RBCs. The patient underwent excision of bilateral cysts under general anesthesia via a sublabial approach. Intraoperatively, smooth, well-circumscribed cystic lesions were visualized, separated by a bony septum (Figure 2).

The cysts were found to be firmly adherent to the underlying mucosa of the nasal cavity floor, being entirely extraosseous with no attachment to the underlying bone. Both cysts were excised and sent separately for histopathological examination.

**Figure 3:**
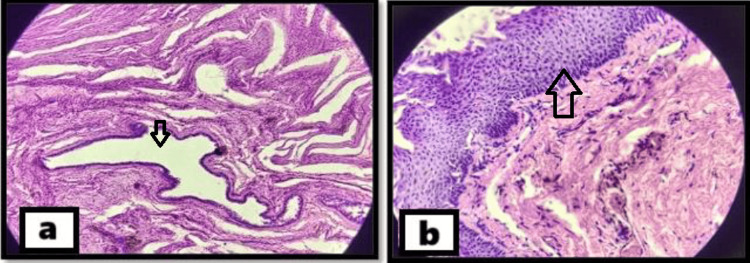
Histopathological images a: the arrow pointing to the cyst; b: the arrow showing stratified squamous epithelium

On microscopic examination, the walls of both cysts showed a lining of ciliated columnar epithelium with interspersed goblet cells. Additionally, the right-sided cyst showed a small focus of benign stratified squamous epithelium, too (Figure 3). The patient was given antibiotics and anti-inflammatory medications in the postoperative period and was followed up at seven days. The patient showed satisfactory postoperative healing.

## Discussion

Although these cysts are developmental in origin, they rarely manifest until adulthood. Bilateral presentation is seen in less than 12% of cases [6]. These cysts most commonly occur between the fourth and fifth decades of life, with incidence in females 3.6 times higher than in males. Currently, it is widely believed that they arise from the residuum of fetal nasolacrimal duct tissue [7]. Clinically, these individuals most commonly present with facial deformities and nasal obstruction. Bimanual palpation of the nasal floor and labial sulcus frequently reveals a soft, fluctuant mass between the upper lip and nasal aperture, which causes elevation of the nasal ala and obliteration of the nasolabial groove. These swellings are usually painless and non-tender unless there is secondary infection or hemorrhage [8].

The CT scan is the preferred imaging modality for evaluation. It displays a uniform, cystic lesion anterior to the piriform aperture. Although useful, MRI is seldom employed in clinical practice [9]. Microscopic examination of nasolabial cysts shows they are often lined by stratified squamous, cuboidal, or pseudostratified columnar epithelium with interspersed goblet and inflammatory cells, as well as collagen deposits [10]. Similar histopathological findings were seen in our patient.

Various other odontogenic and non-odontogenic lesions, such as periodontal cysts, sebaceous cysts, epidermal inclusion cysts, furuncles on the nasal floor, salivary gland neoplasms, premaxillary abscesses, and minor salivary gland neoplasms, can also mimic these cystic lesions. Thus, a definitive diagnosis typically necessitates a correlation of clinical, surgical, radiological, and histological data [11]. The treatment of choice for these cysts is complete surgical enucleation through a sublabial approach. This technique is typically associated with favorable outcomes; however, potential complications may include facial swelling, dental and gingival paresthesia, and wound infection. Transnasal endoscopic marsupialization of the cyst has been described, along with other treatment options, including cauterization, incision and drainage, and sclerosant therapy. However, these techniques have been associated with substantial recurrence rates [12].

## Conclusions

Bilateral nasolabial cysts are extremely rare entities. These lesions are disfiguring for the patient and can also cause nasal obstruction. They may be precipitated by trauma to the face, as seen in this case. This report highlights the importance of considering nasolabial cysts in the differential diagnosis of anterior maxillary swellings, even when lesions are bilateral. Thorough clinical, radiological, and histopathological evaluation is essential for accurate diagnosis and appropriate management, as they may mimic other cystic lesions in the region. Surgical excision is the treatment of choice and offers excellent results.
